# Functional subdivision of the human periaqueductal grey in respiratory control using 7 tesla fMRI

**DOI:** 10.1016/j.neuroimage.2015.02.026

**Published:** 2015-06

**Authors:** Olivia K. Faull, Mark Jenkinson, Stuart Clare, Kyle T.S. Pattinson

**Affiliations:** a FMRIB Centre, Nuffield Department of Clinical Neurosciences, University of Oxford, Oxford, UK; b Nuffield Division of Anaesthetics, Nuffield Department of Clinical Neurosciences, University of Oxford, Oxford, UK

**Keywords:** fMRI, Brainstem, Respiration, Periaqueductal grey

## Abstract

The periaqueductal grey (PAG) is a nucleus within the midbrain, and evidence from animal models has identified its role in many homeostatic systems including respiration. Animal models have also demonstrated a columnar structure that subdivides the PAG into four columns on each side, and these subdivisions have different functions with regard to respiration. In this study we used ultra-high field functional MRI (7 T) to image the brainstem and superior cortical areas at high resolution (1 mm^3^ voxels), aiming to identify activation within the columns of the PAG associated with respiratory control. Our results showed deactivation in the lateral and dorsomedial columns of the PAG corresponding with short (~ 10 s) breath holds, along with cortical activations consistent with previous respiratory imaging studies. These results demonstrate the involvement of the lateral and dorsomedial PAG in the network of conscious respiratory control for the first time in humans. This study also reveals the opportunities of 7 T functional MRI for non-invasively investigating human brainstem nuclei at high-resolutions.

## Highlights

• 7 T MRI was used to image the brainstem during respiratory and motor tasks.
• Breath holds caused deactivation in the lateral and dorsomedial PAG.
• RETROICOR and hypercapnic noise correction techniques were employed.
• We demonstrate the potential of 7 T fMRI to investigate human brainstem respiratory control.

## Introduction

The study of respiratory control is largely focused on the nuclei of the respiratory rhythm generators in the medulla, whilst suprapontine control of respiration is less well understood. The midbrain periaqueductal grey (PAG) is located at the junction of descending efferent commands and ascending sensory information, and has been suggested by animal models to participate within the localised pathway of respiratory response (Kabat et al., 1935; Subramanian, 2012; Subramanian et al., 2008). The human PAG is approximately 14 mm long and 4–5 mm wide (either side of the aqueduct), and almost completely encircles the aqueduct. The PAG has been proposed to be subdivided into four columns on each side; ventrolateral (vlPAG), lateral (lPAG), dorsolateral (dlPAG) and dorsomedial (dmPAG) (Bandler and Shipley, 1994; Dampney et al., 2013; Subramanian, 2012; Subramanian et al., 2008). Direct excitation of these columns in animals has revealed distinct respiratory functions, such as irregular breathing with the vlPAG, prolonged inspirations, expirations and vocalisations from the lPAG, active breathing and tachypnea from the dlPAG, and slow, deep breathing from the dmPAG (Subramanian, 2012; Subramanian et al., 2008).

Whilst animal models allow detailed investigation of functional neuroanatomy, subsequent studies in humans are essential to understand the role of these PAG subdivisions. Human respiratory control networks cannot be assumed to match those derived from animals. Additionally, humans allow the study of *conscious* control of breathing with the addition of subjective feedback, which is not possible in animals. Understanding these respiratory networks is imperative for effective treatment of breathing disorders, such as breathlessness from chronic obstructive pulmonary disease and heart failure (Hayen et al., 2013a; Herigstad et al., 2011), sleep disordered breathing (Morrell et al., 2000), and the dangerous respiratory depression associated with opioid painkillers (Pattinson, 2008).

Functional magnetic resonance imaging (fMRI) is a non-invasive technique that allows high-resolution functional imaging in humans (2–3 mm^3^ voxels at 3 T). The recent introduction of ultra-high-field fMRI at 7 T vastly improves the signal-to-noise ratio of previous imaging, potentiating even higher resolution functional imaging (< 2 mm^3^) and the ability to specifically investigate small nuclei such as the subdivisions of the PAG, previously not possible at 3 T. However, 7 T imaging requires added methodological considerations during both scanning and analysis. Greater B_0_ inhomogeneities at 7 T cause increased distortion and drop-out during echo-planar imaging (EPI), and increases in resolution require longer acquisition times (TR) and cause decreases in temporal signal-to-noise. Additionally, high resolution functional scanning may reveal greater structural and functional differences between individuals, amplifying the importance of image registration for successful group statistical analysis. Therefore, this study aimed to investigate and establish methods to image brainstem centres at 7 T.

Using MRI to investigate respiratory control presents further methodological challenges and requires additional considerations. Arterial pressure of carbon dioxide (PaCO_2_) is a potent vasodilator of cerebral vessels, and thus changes in PaCO_2_ often induced by respiratory challenges confound the blood oxygen level dependent (BOLD) signal (Pattinson et al., 2009a; Pattinson et al., 2009b). Additionally, bulk susceptibility variations in the lungs during the respiratory cycle cause changes in the B_0_ magnetic field, producing physiological noise related to respiratory changes (Glover et al., 2000; Harvey et al., 2008; Raj et al., 2001). Finally, the location of the brainstem close to arteries and pulsating fluid-filled spaces (due to cardiac and respiratory cycles) (Brooks et al., 2013; Cohen et al., 2002) results in a particular susceptibility to physiological noise artefacts, yet it is of great importance as it houses many respiratory control centres.

In this study we used 7 T scanning to investigate the role of the subdivisions of the PAG in short respiratory tasks, taking careful consideration of respiratory imaging confounds. Based on previous work in animals, we hypothesised that BOLD signal changes within the lPAG and dmPAG (associated with prolonged expirations and depressed breathing) would be associated with the inhibitory respiratory tasks of breath holds and vocalisations, but not associated with a simple sensory and motor task.

## Materials and methods

### Subjects

The Oxfordshire Clinical Research Ethics Committee approved the study and volunteers gave written, informed consent. Sixteen healthy, right-handed volunteers (10 males, 6 females; mean age ± SD, 28 ± 7 years) undertook one training session, followed immediately by one MRI scanning session. One subject was excluded from the analysis due to an inability to comply with experimental protocol. Prior to scanning, all subjects were screened for any contraindications to magnetic resonance imaging at 7 T.

### Breathing system

**Fig. 1:**
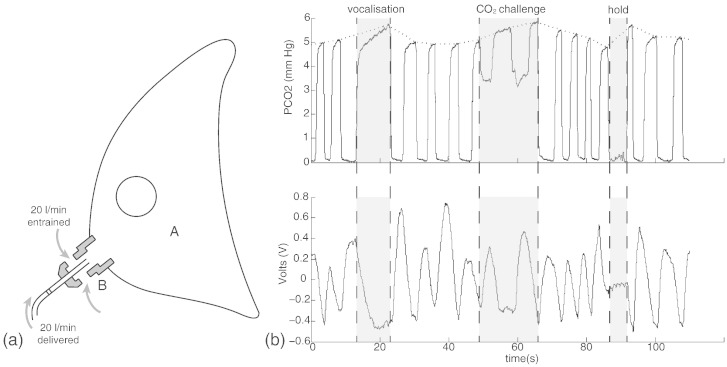
a) Schematic diagram of the venturi mask used in the breathing system. A: Loose plastic venturi mask B: Venturi entrainment device (1:1). b) A section of a respiratory trace from one subject demonstrating the tidal CO_2_ trace (top) and the tidal volume trace from the bellows (bottom). The end-tidal CO_2_ (P_ET_CO_2_) trace was formed by interpolating between the end expiration peaks (dotted line, top trace). The breath hold duration was calculated from the time between the end of expiration CO_2_ trace and the beginning of the subsequent expiration trace, to minimise inclusion of head movement. The vocalisation duration was calculated from the duration between the beginning and end of a vocalisation expiration trace.

A breathing system was used to allow the administration of small CO_2_ challenges mixed with room air, via a venturi entrainment system (Fig. 1a). The CO_2_ challenges were administered to dissociate the changes in global BOLD signal due to changes in arterial PCO_2_ from local BOLD signal changes correlating to activity associated with breath holds and vocalisations (Pattinson et al., 2009a). During scanning, medical air was administered through a loose fitting venturi mask (Ventimask, Intersurgical Ltd, Berkshire, UK) with a 1:1 entrainment ratio of compressed gas:room air. Gas was delivered to the mask at a rate of 20 L/min, and the mask was designed to entrain an equivalent amount of room air. The resulting high gas flow rate delivered by this system (40 L/min) minimises rebreathing of expired gases. The ventimask is loose fitting and therefore considerably more comfortable than a tight fitting mask, but its gas delivery characteristics allows control of end-tidal gases in the volunteer. For the CO_2_ challenges during the functional scan, the medical air was substituted for a CO_2_ mixture (10% CO_2_, 21% O_2_, balance nitrogen) at 20 L/min for periods of 10 s, the entrainment system meant that approximately 5% CO_2_ was delivered to the face mask. The CO_2_ challenges aimed to elevate end-tidal partial pressure of CO2 (P_ET_CO_2_) by approximately 0.8%, to match elevations caused by breath holds and vocalisations.

### Physiological measurements

Physiological measures were recorded continuously during the training session and MRI scan. Chest movements were measured using respiratory bellows surrounding the chest at the approximate level of the 10th rib, and heart rate was measured using a pulse oximeter (9500 Multigas Monitor, MR Equipment Corp., NY, USA). The end-tidal partial pressure of CO_2_ (P_ET_CO_2_) was sampled via nasal cannula (Salter Labs, California, USA) and determined using a rapidly-responding gas analyser (CD-3A; AEI Technologies, Pittsburgh, USA). Subjects were asked to breathe through their nose for the entire experiment. All physiological devices were connected to a data acquisition device (MP150; Biopac Systems Inc., California, USA) coupled to a desktop computer with recording software (Acknowledge 4.2; Biopac Systems Inc., California, USA).

### Magnetic resonance imaging

**Fig. 2:**
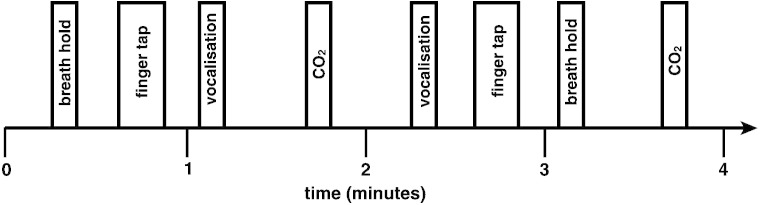
Example four minutes of the BOLD sequence, repeated throughout the acquisition. The order of the breath holds and vocalisations was semi-randomised between the finger opposition and CO_2_ stimuli.

MRI was performed with a 7 T Siemens Magnetom scanner, with 70 mT/m gradient strength and a 32 channel Rx, single channel birdcage Tx head coil (Nova Medical). The fMRI experimental design is illustrated in Fig. 2.

#### Brainstem BOLD scanning

A T2*-weighted, gradient echo EPI functional scan consisted of 333 volumes, and lasted 28 mins and 10 s. The field of view (FOV) comprised 54 coronal–oblique slices of the brainstem and cortex (sequence parameters: TE, 24 ms; TR, 5 s; flip angle, 90°; voxel size, 1 × 1 × 1 mm; GRAPPA factor, 3; echo spacing, 1 ms; slice acquisition order, descending).

#### Structural scanning

A T1-weighted structural scan (MPRAGE, sequence parameters: TE, 2.96 ms; TR, 2200 ms; flip angle, 7°; voxel size, 0.7 × 0.7 × 0.7 mm; inversion time, 1050 ms; bandwidth; 240 Hz/Px) was acquired. This scan was used for registration of functional images, and anatomical overlay of brain activations.

#### Additional scanning

A single volume whole brain EPI was acquired with 128 slices in the same orientation as the functional scan (matched sequence parameters) for registration purposes. Fieldmap scans (sequence parameters: TE1, 4.08 ms; TE2, 5.1 ms; TR, 620 ms; flip angle, 39°; voxel size, 2 × 2 × 2 mm) of the B_0_ field were also acquired in the same orientation to assist distortion-correction of scans.

### Stimuli and tasks

During the BOLD scan, subjects performed 14 repeats of the following tasks: expiratory breath hold, vocalisation and finger and thumb opposition task (described to subjects as “finger tapping”). This paradigm was (adapted from previous breath hold research (McKay et al., 2008; Pattinson et al., 2009a)). Cues for these tasks were presented on the screen as the words ‘HOLD’, ‘SING’ and ‘TAP’ (‘REST’ was displayed for the remainder of the experiment), in white letters on a black background. During the training session, the subjects had a chance to practice each of the tasks under observation. The instructions for the breath hold were to stop breathing at the end of the current breath, maintaining the hold until the ‘HOLD’ cue was exchanged for ‘REST’. At the termination of the breath hold, the subject was asked to breathe out any remaining air in their lungs for an end-tidal CO_2_ reading. The instructions for the vocalisation were to produce a closed-mouth single note from the top of the next breath, for the duration of the ‘SING’ cue. Both the ‘HOLD’ and ‘SING’ cues were presented for a duration of 10 s; therefore the tasks were each less than 10 s. The finger opposition task consisted of an opposition movement conducted with the right hand, and the ‘TAP’ cue was presented for 15 s. It is well known that the cuneate nucleus of the medulla is a sensory nucleus in the fine touch and proprioception pathway, prior to decussation (Craven, 2011). Therefore, activation of the ipsilateral cuneate associated with the finger opposition task was used by Pattinson et al. (2009a), as a functional localiser to provide confidence in the precision of brainstem registrations. We repeated this functional localiser task in the present study, to validate our methodology and assure the accuracy of the registration of the 1 mm^3^ functional images. Each of the three tasks was repeated 14 times within the BOLD scan of each subject.

The respiratory stimuli of breath holding and vocalisations both cause an increase in arterial PCO_2_ (hypercapnia), which leads to vasodilation of the cerebral vessels and an increase in blood flow (Cohen et al., 2002). Hypercapnia increases the grey matter BOLD signal in correlation with the local BOLD signal of interest from these stimuli. Therefore, to decorrelate the effects of hypercapnia from the localised BOLD responses correlating with breathing control, additional, repeated CO_2_ challenges were interspersed during rest periods in the protocol. This means that the timecourse of the CO_2_ is different to the time course of the breath holds. A CO_2_ regressor was then created by extrapolating between end-tidal peaks, and this trace was entered into the general linear model as a physiological regressor of no interest (described in detail in Pattinson et al., 2009a).

### Analysis

#### Preprocessing

Image preprocessing was performed using the Oxford Centre for Functional Magnetic Resonance Imaging of the Brain Software Library (FMRIB, Oxford, UK; FSL version 6.0; http://www.fmrib.ox.ac.uk/fsl/). The following processing methods were used prior to statistical analysis: motion correction (MCFLIRT; Jenkinson et al., 2002), removal of the nonbrain structures (skull and surrounding tissue) (BET; Smith, 2002; Woolrich et al., 2001), spatial smoothing using a full-width half-maximum (FWHM) Gaussian kernel of 2 mm, and high-pass temporal filtering (Gaussian-weighted least-squares straight line fitting; 120 s cutoff period). B_0_ field unwarping was conducted with a combination of FUGUE and BBR (Boundary-Based-Registration; part of FEAT: FMRI Expert Analysis Tool, version 6.0) tools (Greve and Fischl, 2009; Jenkinson et al., 2002), and the functional scans were corrected for cardiac- and respiratory-related noise with RETROICOR (Brooks et al., 2008; Glover et al., 2000; Greve and Fischl, 2009; Harvey et al., 2008). Time-series statistical analysis was performed using FILM, with local autocorrelation correction (Andersson et al., 2007; Woolrich et al., 2001).

#### Image registration

Careful attention was paid to image registration, as the finer resolution afforded by 7 T MRI requires greater registration accuracy for group statistics to be possible. After preprocessing, the functional scans were registered to the MNI152 (1 mm^3^) standard space [average T1 brain image constructed from 152 normal subjects at the Montreal Neurological Institute (MNI), Montreal, QC, Canada] using a three-step process.

• Linear registration (FLIRT) with 6 degrees of freedom (DOF) was used to align the partial FOV scan to the whole-brain EPI image (Jenkinson et al., 2002; Worsley, 2001).
• Registration of subjects' whole-brain EPI to T1 structural image was conducted using BBR (6 DOF) with (nonlinear) fieldmap distortion-correction (Devonshire et al., 2012; Greve and Fischl, 2009).
• Registration of the subjects' T1 structural scan to 1 mm standard space was performed using an affine transformation followed by nonlinear registration (FNIRT) (Andersson et al., 2007; Handwerker et al., 2004).

#### Voxelwise analysis

fMRI data processing was performed using FEAT (FMRI Expert Analysis Tool), version 6.0, part of FSL (FMRIB's Software Library; www.fmrib.ox.ac.uk/fsl). The first-level analysis in FEAT incorporated a general linear model, where the finger opposition regressor was derived from the protocol timing values by convolution with an HRF basis set (see below). The two respiratory stimuli (breath holds and vocalisations) timings were modelled from the respiratory physiological traces (Fig. 1b). P_ET_CO_2_ was included as an additional regressor, de-correlating the CO_2_-induced BOLD changes from the respiratory stimuli throughout the functional scan. This trace was formed by linearly interpolating between the expired CO_2_ peaks. This technique assumed a linear rise in P_ET_CO_2_ throughout a breath hold, as values were only available for the breath immediately before and at the end of each hold. Previous research has indicated that variations in the haemodynamic response function (HRF) are apparent throughout the brainstem and cortex (Devonshire et al., 2012; Woolrich et al., 2004b), and between subjects (Handwerker et al., 2004; Smith and Nichols, 2009). To account for possible changes in the HRF, including slice-timing delays, we used an optimal basis set of three waveforms (FLOBS: FMRIB's Linear Optimal Basis Sets, default FLOBS supplied in FSL (Woolrich et al., 2004a)), instead of the standard gamma waveform. This models the changes induced by altered HRFs or slice-timing, but does induce some bias into the estimation of the main effect size. This bias takes the form of an underestimation of the effect size, which is a conservative error that affects the statistical power, and therefore will not inflate the false positive rate. The second and third FLOBS waveforms, which model the temporal and dispersion derivatives, were orthogonalised to the first waveform, of which the parameter estimate was then passed up to the higher level to be used in group analysis.

Voxelwise statistical analysis using basis functions was extended to a group level, in a mixed-effects analysis using FLAME (FMRIB's Local Analysis of Mixed Effects) (Woolrich et al., 2004b). *Z* statistic images were thresholded using clusters determined by *Z* > 2.3 and a (corrected) cluster significance threshold of p < 0.05. A small-volume-corrected analysis of the PAG (as our a-priori area of interest; mask = 698 voxels) was then conducted, using threshold-free cluster enhancement (TFCE) corrected for family-wise error. TFCE provides an alternative method to enhance cluster-like structures in an image, without a pre-determined initial cluster-forming threshold (Smith and Nichols, 2009). TFCE produces an output where voxel-wise values represent the amount of cluster-like local support, illustrating the significance of voxels within a cluster rather than the significance of the cluster as a whole whilst maintaining strict false positive control by using permutation-based family-wise-error correction (Smith and Nichols, 2009).

## Results

### Physiology

Group average heart rate (± SD) during the brainstem BOLD scanning was 65 (± 11) beats per minute. An example respiratory trace is given in Fig. 1b. Baseline P_ET_CO_2_ (± SD) was 4.7% (± 0.7%), with breath holds increasing P_ET_CO_2_ by 0.8% (± 0.2%) and 0.6% (± 0.2%) with vocalisations. CO_2_ challenges induced an average increase in P_ET_CO_2_ of 0.9% (± 0.1%).

### Periaqueductal grey analysis

**Fig. 3:**
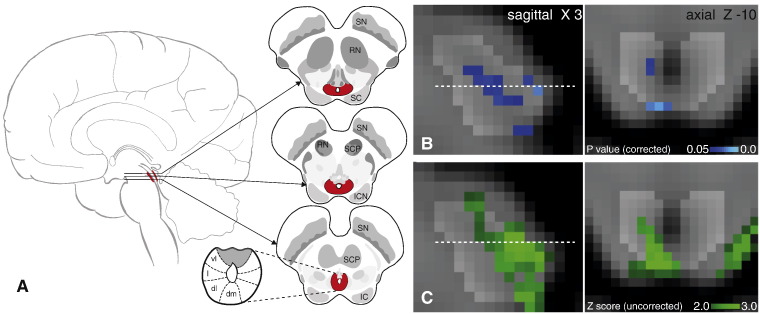
Periaqueductal grey (PAG) response to breath hold. A. Representation of the location of the PAG within the brain, three sagittal slices and the current opinion of the subdivisions of the PAG. B. Localisation of the functional decreases in BOLD signal within the PAG (p < 0.05; small-volume-corrected for multiple comparisons using overlaid PAG mask), where the images consist of a colour-rendered statistical map superimposed on a standard (MNI 1 mm^3^) brain. Dashed line represents Z-10 location. C. Uncorrected Z score image of PAG deactivation from whole brain analysis, prior to masking. Abbreviations: SN, substantia nigra; RN, red nucleus; SC, superior colliculus; SCP, superior cerebellar peduncle; ICN, inter-colliculi nucleus; IC, inferior colliculus; vl, ventrolateral PAG, l, lateral PAG; dl, dorsolateral PAG; dm, dorsomedial PAG.

The results from a small-volume family-wise-error corrected analysis of the PAG revealed significant (p < 0.05) deactivation in two areas correlating with the breath hold task (Fig. 3). One of these deactivation clusters followed the lateral column on the right side of the PAG (12 voxels), and the second was located in the right caudal dorsolateral PAG (8 voxels). Uncorrected z scores within the PAG are also presented in Fig. 3, demonstrating the deactivations extending to the inferior border of the PAG. Column locations were defined using tractography results from a recent diffusion tensor imaging study (Ezra et al., ISMRM abstract, 2014). No significant activations or deactivations were found in the PAG for either vocalisation or the finger opposition task.

### Cortex

**Fig. 4:**
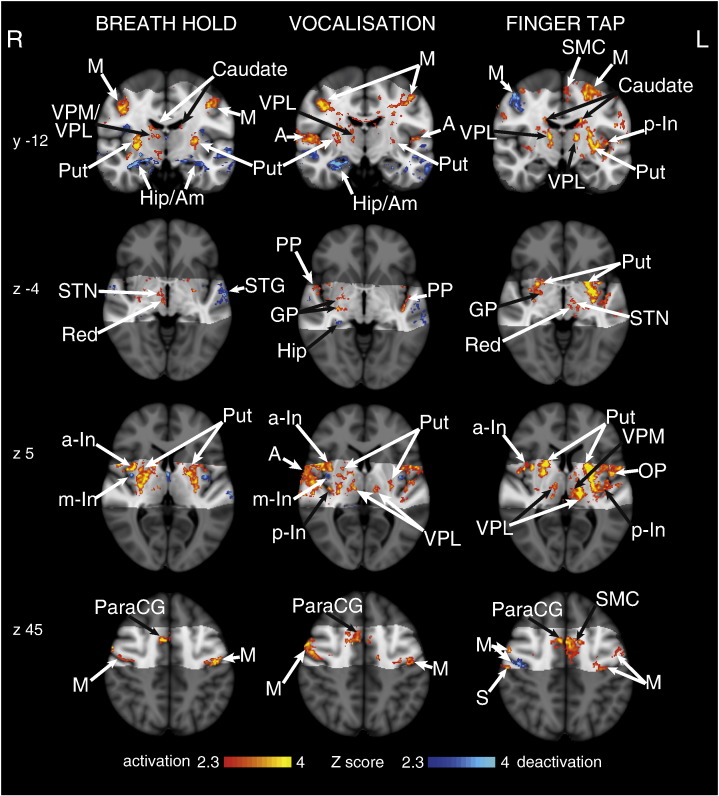
BOLD response to breath holds, vocalisations and finger opposition in 15 subjects, having accounted for CO_2_-induced vasodilation. The images consist of a colour-rendered statistical map superimposed on a standard (MNI 1 mm^3^) brain. The bright grey region represents the coverage of the coronal-oblique functional scan. Significant regions are displayed with a threshold *Z* > 2.3, with a cluster probability threshold of p < 0.05 (corrected for multiple comparisons). Abbreviations: M, motor cortex; Caudate, caudate nucleus; SMC, supplementary motor cortex; Put, putamen; A, auditory cortex; Hip, hippocampus; Am, amygdala; thalamic nuclei: VPM, ventral posteromedial nucleus; VPL, ventral posterolateral nucleus; MDN, medial dorsal nuclei; a-In, anterior insula; m-In, middle insula; p-In, posterior insula; PP, planum polare; STN, subthalamic nucleus; Red, Red nucleus; STG, superior temporal gyrus; GP, globus pallidus; OP, operculum; S, post central gyrus (sensory cortex); paraCG, paracingulate gyrus; activation, increase in BOLD signal; deactivation, decrease in BOLD signal. R (right) and L (left) indicate image orientation.

We observed significant signal increases bilaterally in the motor cortex, supplementary motor cortex, anterior cingulate (ACC) and paracingulate cortices, primary sensory cortex, anterior insula and putamen (Fig. 4). Breath holds also correlated with bilateral BOLD signal increases in the supramarginal gyrus and caudate nucleus, whilst vocalisations correlated with right side supramarginal gyrus activation. Vocalisations were also associated with thalamic activation bilaterally in the VPL nucleus, Heschl's gyrus (primary auditory cortex) and the planum polare. Conversely, breath holds were associated with right side BOLD signal increases in the thalamus [ventral posterolateral (VPL) and ventral posteromedial (VPM) nuclei], subthalamic nucleus and red nucleus.

### CO_2_ challenges

The hypercapnia challenges and the resultant CO_2_ regressor produced strong BOLD signal increases throughout the grey matter of the brain. Furthermore, increases in BOLD signal correlating to the CO_2_ regressor were observed within the PAG, localised to the grey matter and excluding the aqueduct (supplementary Fig. 1).

### Finger opposition task

**Fig. 5:**
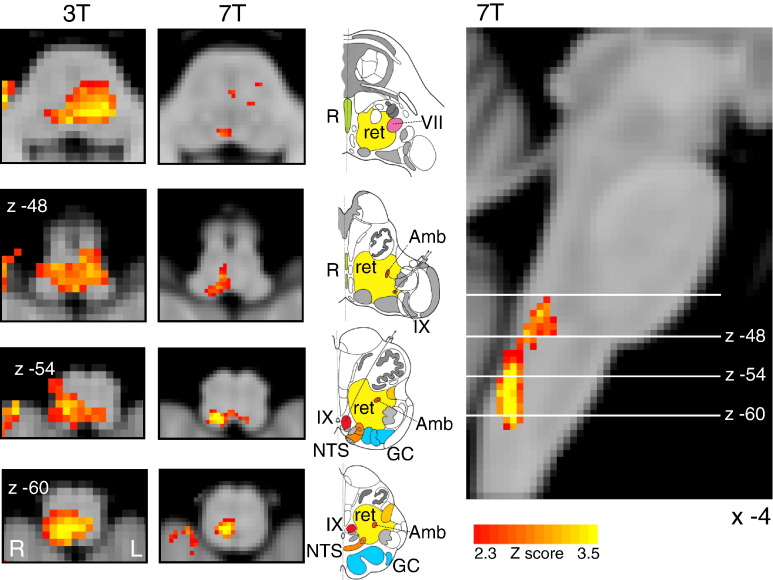
Demonstration of the use of finger opposition as a functional localiser in brainstem fMRI at 3 T and at 7 T, by imaging activation in the ipsilateral cuneate nucleus of the medulla (z -54). The 3 T data is derived from previously-published results (Pattinson et al., 2009a). This technique provides confidence in the analysis model and registration accuracy of the current 7 T study. The images consist of a colour-rendered statistical map superimposed on a standard (MNI 1 mm^3^) brain. Significant regions are displayed with a threshold Z > 2.3, with a cluster probability threshold of p < 0.05 (corrected for multiple comparisons). The sagittal image on the right displays the position of slices, for clarity only displayed from the 7 T acquisition. Abbreviations: R, raphe nuclei; ret, nuclei reticularis; VII, facial nucleus; Amb, nucleus ambiguous; IX, glossopharyngeal nucleus; NTS, nucleus tractus solitaries; GC, gracile (medial) and cuneate (lateral) nuclei (in blue). R (right) and L (left) indicate image orientation. Original line drawings adapted from Duvernoy (1995).

Finger opposition resulted in significant signal increases bilaterally in the motor cortex (more extensive activation in the contralateral left motor cortex), supplementary motor cortex, anterior cingulate (ACC) and paracingulate cortices, primary sensory cortex, anterior insula cortex, operculum, caudate nucleus and putamen (supplementary Fig. 1). Bilateral signal increases were seen in the thalamic VPL nuclei, as well as the left thalamic VPM nucleus. In addition, activations were observed in the left subthalamic and red nuclei, left pons, right (ipsilateral) cuneate nucleus of the medulla (Fig. 5), and bilateral cerebellum (VI and VIIIa lobules).

## Discussion

### Main findings

Assuming that the human PAG has a columnar structure similar to that in animals, and corresponding with recent human neuroimaging findings (Ezra et al., 2014 ISMRM abstract; paper under review), we have identified respiratory-related activity that appears to be localised within the lateral and caudal dorsomedial columns of the PAG. Cortical activity associated with breath holding was consistent with previous results (Cowie and Holstege, 1992; Pattinson et al., 2009b; McKay et al., 2008), and highly localised within cortical regions. As expected, with the control sensory and motor task of finger opposition, BOLD activity was identified within the ipsilateral cuneate nucleus in the medulla, a sensory nucleus processing fine touch and proprioception, validating our methodology.

### PAG and respiratory control

This study has demonstrated differential activity localised within the columns of the PAG (lPAG and dmPAG), correlating with the respiratory task of a breath hold. In comparison, no observable differences in any areas of the PAG were found with a respiratory vocalisation task, nor with a motor finger-tapping task. This study is the first to functionally localise respiratory activity within the human PAG, with sufficient resolution (1 mm^3^) to have confidence in the positions of activations in relation to the current theory of columnar subdivisions. Using a Gaussian kernel of 2 mm (FWHM) for spatial smoothing, blurring of the BOLD signal changes within the PAG was minimised. The deactivation seen within the lPAG, in particular, follows a columnar structure that is consistent with previous work from animal models.

Whilst there have been many neuroimaging studies reporting involvement of the PAG (Linnman et al., 2012), the function(s) of the PAG have predominantly been linked to pain, anxiety, bladder and bowel control, and cardiovascular regulation (Linnman et al., 2012). Whilst there has been some implications of PAG involvement in respiratory control (Corfield et al., 1995; Pattinson et al., 2009a; Topolovec et al., 2004) this research has been limited by functional resolution, extensive smoothing, or registration issues that have impeded group-level analysis, making accurate localisation to the PAG or its subdivisions impossible. In addition, significant results within the PAG have been found using statistics uncorrected for multiple comparisons (Mobbs et al., 2007), and a more recent 7 T study used a column segmentation of the PAG that is inconsistent with animal literature, and masking of the cerebral aqueduct to dissociate activity within PAG columns (Satpute et al., 2013). Therefore, the results of this study demonstrate the potential for 7 T MRI to be used to investigate the roles of the subdivisions of the PAG within respiratory control using robust statistical methodology, when careful attention is paid to registration and noise correction of functional data.

Deactivation within the PAG was localised to the lateral column and caudal section of the dorsomedial column, (Ezra et al., ISMRM abstract, 2014). Activity in the lateral and dorsomedial columns of the PAG during inhibition of ventilation is consistent with previous respiratory control experiments in animals, where these columns have been associated with depressed ventilatory behaviours such as prolonged inspirations and expirations (lPAG), and slow, deep breathing (dmPAG). Conversely, the vlPAG and dlPAG have been associated with more active and irregular ventilation in animals (Subramanian, 2012; Subramanian et al., 2008). Whilst excitation of the vlPAG and lPAG in decerebrate cats has previously produced vocalisations of mews and hisses (Subramanian et al., 2008), we did not see activation in any of the PAG columns during vocalisations. It is possible that the vocalisation pathway within the cortex and brainstem is not consistent from animals to humans, or possibly that brainstem movement during vocalisations masked these activations in the current experiment.

Whilst one cannot determine from the BOLD signal whether the deactivations in the PAG represent either changes in motor drive or incoming sensory information, it has been established that the lateral PAG receives somatotopically organised spinal sensory afferents (Craven, 2011; Keay and Bandler, 2001) and could thus be involved in monitoring ventilatory feedback from the chest. Conversely, evidence exists for direct descending connections from the lateral and dorsomedial PAG to the midline medulla (Cowie and Holstege, 1992), demonstrating the potential for involvement in descending motor commands to respiratory centres in the medulla. Therefore, it is possible that the lPAG is involved with monitoring respiratory sensations from the chest as they ascend up to higher cortical areas of sensation or, together with the dmPAG, in the descending motor drive for changes in respiration.

A recent study used diffusion tensor imaging (DTI) to segment the human PAG (Ezra et al., 2014 ISMRM abstract; paper under review), and found a similar four-column structure with animal models of the PAG (Subramanian, 2012; Subramanian et al., 2008). Ezra et al. (2014) revealed strong connectivity between the lateral column of the PAG and the primary motor and sensory cortices in humans, which supports our hypothesis of the potential role of the lPAG in either respiratory sensation or in the descending motor drive for changes in ventilation. Further research combining functional and structural techniques (such as DTI) may help to advance our understanding of the role of the subdivisions of the PAG in human respiratory control.

The secondary findings of this study were that cortical activations associated with breath holding at 7 T largely agree with previous breath hold research at 3 T (Feinberg et al., 2010; Pattinson et al., 2009a; McKay et al., 2008), with a higher degree of localisation. It is possible that the cortical results were underestimated, due to slice-timing differences across the field of view with a 5 second TR. However, despite the possibly conservative nature of these results, the cortical consistency displayed with short 10 second breath holds, and additional deactivation of the lPAG and dmPAG, suggests that these PAG areas are part of the conscious breathing control network that is invoked during voluntary cessation of breathing.

### Finger opposition task

As well as a control motor task, the finger opposition task was used as a robust comparison between ultra-high field and lower field fMRI studies, for confidence in analysis techniques and subsequent interpretation of results. Importantly, we had a firm a priori hypothesis that localised BOLD signal increase would be seen in the ipsilateral cuneate nucleus of the medulla, which is a sensory nucleus in the fine touch and proprioception pathway (Craven, 2011). This activation was consistent with previous findings at 3 T (Fig. 5), and demonstrates the registration accuracy within the brainstem to allow activations within small nuclei to survive group analysis. Therefore, medullary motor activity was used to validate the registration and modelling in the current study. However, medullary and pontine activations previously seen with breath holds at 3 T (Pattinson et al., 2009a; McKay et al., 2008) were apparent subthreshold, but did not survive multiple comparison correction, possibly due to the reduction in signal experienced when voxel size is reduced to 1 mm^3^.

### Scanning and analysis techniques

Whilst the use of ultra-high field MRI at 7 T permits improved spatial resolution in functional imaging, it is accompanied by a compounding of many of the challenges experienced with lower resolution imaging. The use of 7 T imaging increases intrinsic voxel signal compared to 3 T, whilst decreasing the voxel size to 1 mm^3^ reduces this signal, requiring longer scan acquisitions to amass adequate statistical power. Furthermore, acquiring 1 mm^3^ voxels requires a longer repetition time (TR) compared to larger voxels for the same brain coverage. To limit the TR to 5 s we restricted our field of view to a coronal–oblique slice, to collect enough time points within the functional scan for adequate temporal SNR. Restricting the field of view may compromise wider brain network analysis, and techniques such as parallel imaging (Arthurs and Boniface, 2002; Feinberg et al., 2010) may allow broader brain coverage in future high-resolution studies.

Brain imaging of respiratory tasks can be challenging due to stimulus-correlated noise, such as induced changes in blood gases and possible task-related head movement. To address these issues, the duration of the respiratory tasks in this study was limited to 10 s to minimise the changes in PaCO_2_ and movement. The resulting unavoidable small changes in PaCO_2_ were dissociated from the BOLD signal of the respiratory stimuli using short periods of additional CO_2_ (10% CO_2_ in air). However, the long TR (5 s) reduced statistical power by limiting the number of data points collected within each stimulus. This could be addressed in future studies by reducing the TR by either further decreasing the field of view, increasing the voxel size, or using parallel imaging techniques (Feinberg et al., 2010), which would allow more measured volumes within a short respiratory task.

Previous research has shown a widespread decrease in BOLD signal with breath holds, proposed as a result of vascular effects (Thomason et al., 2005). However, as can be seen in Fig. 4, this study found differential activation and deactivation, rather than a global decrease in BOLD signal correlating with breath holds. There are some key differences in techniques and analysis methods that may explain the possible discrepancies between the current study and previous fMRI investigations. Firstly, the breath hold used in the current study is an end-of-breath hold, which produces a different chest diameter in the scanner bore, and will result in differences in intrathoracic pressures and physiological noise in the B_0_ field. Further, Thomason et al. (2005) postulated that the decrease in BOLD signal resulted from a decrease in intrathoracic pressure and cerebral blood flow (CBF), whilst a recent paper by Hayen et al. (2013b) showed that a decrease in intrathoracic pressure (whilst maintaining stable end-tidal CO_2_) actually resulted in a small increase in CBF. Additionally, Thomason et al. (2005) did not report any measurements of end-tidal CO_2_, and thus the effect of hypercapnia in this study cannot be fully explored. Therefore, whilst there are significant challenges in dissociating task-specific fMRI signal from residual vascular effects of breath holds, we feel the techniques used in the current study have minimised the influence of this task-related noise.

A particular problem with brainstem fMRI is the inherently low signal to noise compared to cortical areas (Brooks et al., 2013; Devonshire et al., 2012; Harvey et al., 2008). The brainstem suffers from a larger influence of physiological noise, necessitating the use of either retrospective imaging techniques such as RETROICOR (RETROspective Image CORrection; Glover et al., 2000; Harvey et al., 2008), or data-driven approaches such as independent component analysis (ICA, reviewed in Brooks et al., 2013) to correct for cardiac and respiratory artefacts. RETROICOR was used in the current study, but as no formal comparison has been made between retrospective correction and ICA techniques, future work may benefit from ICA analysis or even a combination of both.

A current problem with brainstem imaging is at the existence of greater static B_0_ field inhomogenieties compared to many areas of the cortex, due to proximity to bone and air-filled cavities. In this study significant signal loss was often seen in areas such as the anterior pons, although the degree of dropout varied between individuals. Whilst we did not explore the role of pontine nuclei in this study, future investigations may need to address this issue for further brainstem imaging.

Finally, special care was taken during the registration of functional images in this study. Whilst greater details of cerebral structures in functional scans are able to be visualised with 1 mm^3^ voxels, so too are the differences seen between individual brains, between hemispheres of a cerebrum or even between sides of a brainstem. Therefore, registration of functional scans through to the standard brain must be extremely accurate to allow reflective group statistics, particularly for smaller structures such as those within the brainstem.

Whilst this study has shown that investigation of small brainstem nuclei is possible using ultra-high field fMRI, future research would greatly benefit from incorporating multiband echo planar imaging acquisition strategies (Moeller et al., 2010). This would reduce the TR, allowing more time points, greater brain coverage or even finer resolution scanning, for more detailed explorations into brainstem respiratory nuclei such as the PAG.

## Conclusions

Imaging the brainstem using 7 T MRI is known to be extremely challenging. However, with rigorous physiological noise modelling, consideration of different HRF within the brainstem, and careful attention to distortion minimisation, we have successfully imaged respiratory-related activity in distinct columns of the PAG. In particular, reductions in the BOLD signal within the lPAG and dmPAG were associated with short breath holds, consistent with animal research. Therefore, these results indicate that the lateral and dorsomedial PAG columns are activated as part of the volitional respiratory control network, and are involved with either the motor inhibition, or sensation of depressed ventilation, or both. This study demonstrates that 7 T MRI can successfully be used for functional investigations into brainstem respiratory nuclei, and that the columns of the PAG may have an important role within the respiratory control network in humans.
