# Supplementary material for: Functional subdivision of the human periaqueductal grey in respiratory control using 7 tesla fMRI
Source: Neuroimage. 2015 Jun;113:356–64. doi: 10.1016/j.neuroimage.2015.02.026 (PMC4441042; doi:10.1016/j.neuroimage.2015.02.026)
Supplement: Supplementary file 1 — Supplementary material. [file mmc1.pdf]

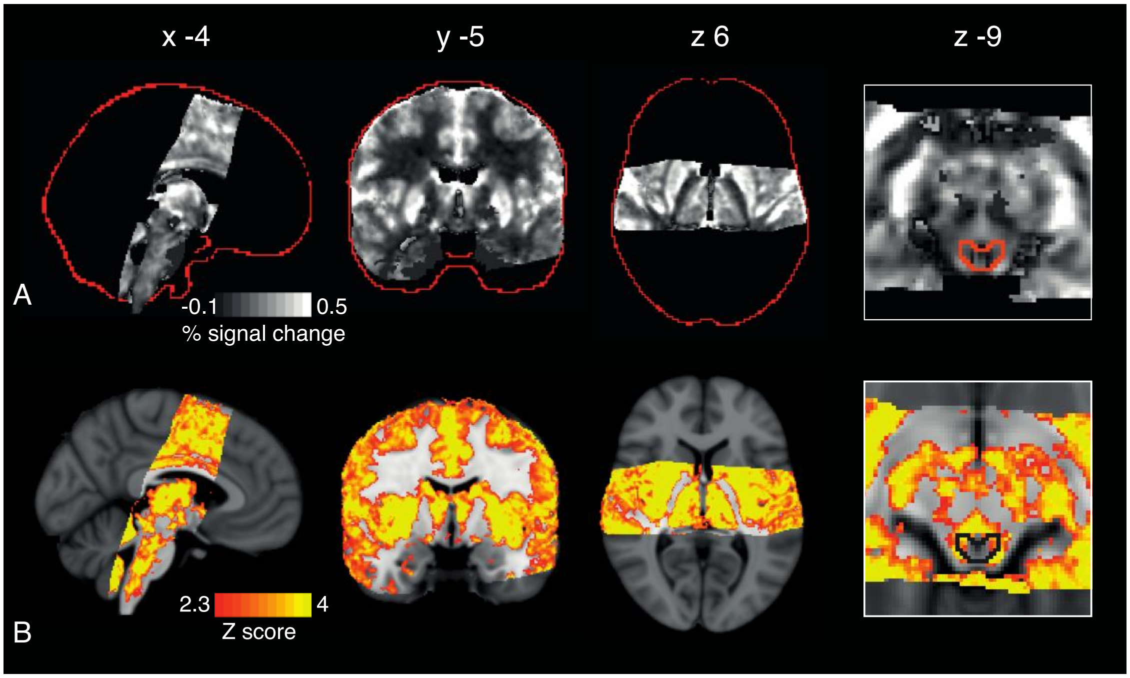

Supplementary Figure 1. A. Global BOLD signal change correlating with changes in end tidal carbon dioxide ($P_{ET}CO_2$). The red lines indicate the edges of the the brain, derived from the MNI ($1mm^3$) standard brain. Image on the right is a zoom to show signal changes within the PAG (outlined in red). B. Statistically significant generalised grey matter signal increases as a result of hypercapnia. PAG outlined in black on the far right. A carbon dioxide (CO_2) trace was created by extrapolating between end-tidal CO_2 peaks, and small hypercapnic challenges were administered during rest periods to dissociate hypercapnic effects from respiratory challenges. The images consist of a colour-rendered statistical map superimposed on a standard (MNI $1mm^3$) brain. Significant regions are displayed with a threshold $Z > 2.3$, with a cluster probability threshold of $p < 0.05$ (corrected for multiple comparisons).

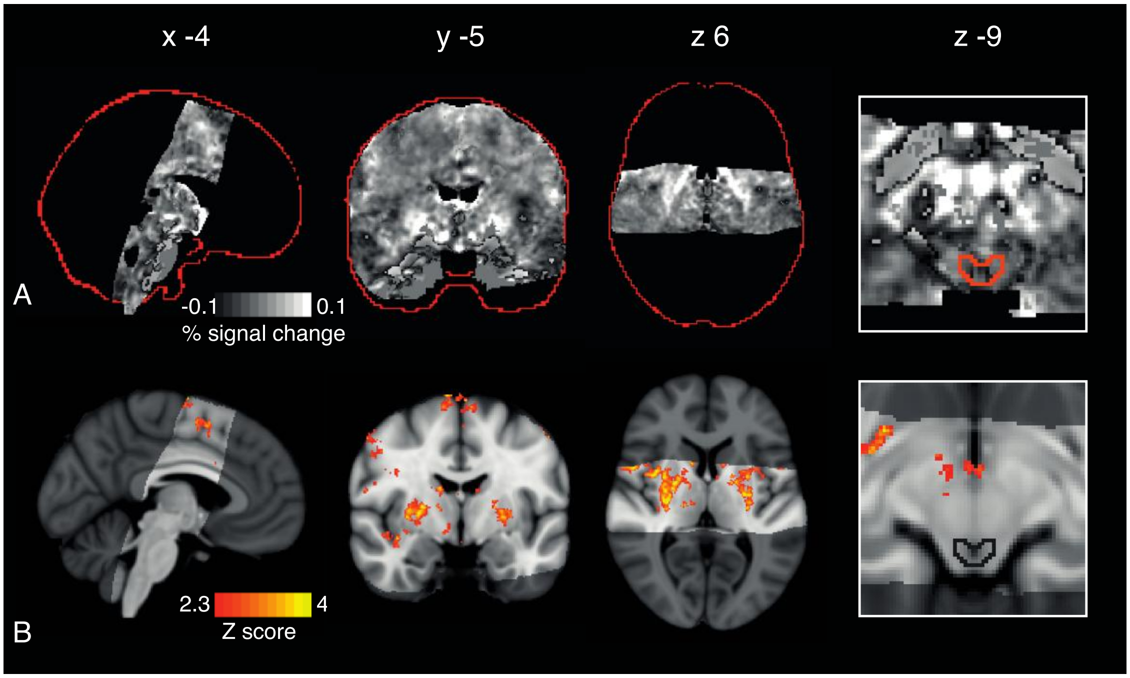

Supplementary Figure 2. A. Global BOLD signal change correlating with breath holds. The red lines indicate the edges of the brain, derived from the MNI ($1mm^3$) standard brain. Image on the right is a zoom to show signal changes within the PAG (outlined in red). B. Statistically significant signal increases as a result of breath holds. PAG outlined in black on the far right. The images consist of a colour-rendered statistical map superimposed on a standard (MNI $1mm^3$) brain. Significant regions are displayed with a threshold $Z > 2.3$, with a cluster probability threshold of $p < 0.05$ (corrected for multiple comparisons).

Locations of signal maxima in response to breath hold regressor								
Region	left				right			
	x	y	z	max Z score	x	y	z	max Z score
Activations								
Motor cortex	-59	4	20	4.64	51	9	22	5.34
Motor cortex	-43	-15	43	4.28	44	-13	40	4.20
Putamen	-20	-2	5	4.79	29	-14	2	4.83
Anterior Insula	-40	5	1	3.27	40	6	2	4.57
Cingulate cortex	-1	14	30	3.00	3	13	29	3.38
Paracingulate cortex	-4	8	48	3.03	7	8	45	5.02
Supramarginal gyrus	-59	-24	26	3.54	63	-22	26	4.89
Sensory cortex	-42	-17	43	4.55	43	-11	36	4.27
Supplementary MC	-5	6	54	3.82	3	7	64	4.30
Caudate nucleus	-17	-16	20	3.92	15	-9	20	3.77
Thalamus					11	-16	4	3.82
Thalamus					9	-14	11	4.11
Subthalamic nucleus					8	-11	-7	3.45
Red nucleus					6	-19	-4	3.26
Deactivations								
PAG, lateral*					3	-33	-11	P=0.032
PAG, dorsomedial*					2	-37	-10	P=0.014
Middle Insula	-39	-6	13	3.97	39	-3	5	3.90
Hippocampus	-32	-20	-15	3.70	30	-13	-15	4.14
Parahippocampal gyrus	-26	-35	-15	3.43	27	-32	-16	3.87
Amygdala	22	-7	-11	4.25	-26	-6	-20	3.72
Pons	-5	-26	-27	3.80				
Pons	-4	-37	-36	3.35				

Cerebellum					2	-51	-46	4.31

Supplementary Table 1. Co-ordinates of local maxima of significant increases (activations) and decreases (deactivations) in the BOLD response to breath holding. Values derived from cluster-based analysis. The most significant maximum is listed for each anatomical location. Co-ordinates are in mm in standard space of MNI ($1mm^3$). x, distance right (+) or left (-) of the mid saggital line; y, distance anterior (+) or posterior (-) from a vertical plane through the anterior commissure; z, distance above (+) or below(-) the intercommisurial plane. Abbreviations: MC, motor cortex; PAG, periaqueductal gray. *PAG analysis was conducted using threshold free cluster enhancement, corrected for multiple comparisons within the PAG, producing P values representing cluster-like local support.
